# Supplementary material for: Concordance of Non-Alcoholic Fatty Liver Disease and Associated Factors among Older Married Couples in China
Source: Int J Environ Res Public Health. 2023 Jan 12;20(2):1426. doi: 10.3390/ijerph20021426 (PMC9859299; doi:10.3390/ijerph20021426)
Supplement: Supplementary file 1 [file ijerph-20-01426-s001.zip › ijerph-2037497-supplementary.pdf]

SUPPLEMENTAL MATERIAL

Concordance of Non-Alcoholic Fatty Liver Disease and Associated Factors among Older Married Couples in China

Xueli Yuan [1,†], Wei Liu [2,†], Wenqing Ni [1], Yuanying Sun [1],

Hongmin Zhang [1], Yan Zhang [1], Peng Yin [2,*] and Jian Xu [1,*]

Table S1. The distribution of the comorbidities among 34,704 NAFLD patients.

Diseases	Overall, N (%)	Husband, N(%)	Wife, N (%)
NAFLD	34,704 (100)	14,622 (100)	20,082 (100)
Comorbidities			
Hypertension	21,856 (63.0)	9,404 (64.3)	12,452 (62.0)
Diabetes	10,734 (30.9)	4,646 (31.8)	6,088 (30.3)
Hyperlipidemia	19,280 (55.6)	8,365 (57.2)	10,915 (54.4)
Any of three diseases	29,795 (85.9)	12,724 (87.0)	17,071 (85.0)

Abbreviations: NAFLD: non-alcoholic fatty liver disease.
